# The COVID-19 Pandemic, Rising Inflation, and Their Influence on Dining Out Frequency and Spending

**DOI:** 10.3390/nu15061373

**Published:** 2023-03-12

**Authors:** Jingjing Gao, Odessa E. Keenan, Abbey S. Johnson, Carissa A. Wilhelm, Rajib Paul, Elizabeth F. Racine

**Affiliations:** 1 Texas A&M AgriLife Research, Texas A&M University, 1380 A&M Circle, El Paso, TX 79927, USA; 2 Texas A&M AgriLife Extension Service, Texas A&M University, 17360 Coit Road, Dallas, TX 75252, USA; 3 Department of Public Health Sciences, University of North Carolina at Charlotte, 9201 University City Blvd, Charlotte, NC 28223, USA

**Keywords:** diet quality, COVID-19, inflation rate, restaurant, food cost, dining out

## Abstract

Background: High intake of food away from home is associated with poor diet quality. This study examines how the COVID-19 pandemic period and Food Away from Home (FAFH) inflation rate fluctuations influenced dining out behaviors. Methods: Approximately 2800 individuals in Texas reported household weekly dining out frequency and spending. Responses completed prior to the COVID-19 pandemic (2019 to early 2020) were compared to the post-COVID-19 period (2021 through mid-2022). Multivariate analysis with interaction terms was used to test study hypotheses. Results and Conclusion: From the COVID-19 period (before vs. after), the unadjusted frequency of dining out increased from 3.4 times per week to 3.5 times per week, while the amount spent on dining out increased from $63.90 to $82.20. Once the relationship between dining out (frequency and spending) was adjusted for FAFH interest rate and sociodemographic factors, an increase in dining out frequency post-COVID-19 remained significant. However, the unadjusted increase in dining out spending did not remain significant. Further research to understand the demand for dining out post-pandemic is warranted.

## 1. Introduction

Food purchased away from home (FAFH) is often associated with higher calories, sodium, and fats and a lower healthy-eating index [1]. Over the past 35 years, Americans have increased the proportion of their food dollars spent on FAFH, specifically, from 44% in 1987 (ERS-EIB-196) to 55% in 2019 [2]. This increase in FAFH spending and the tendency for FAFH to be less healthy has led to an increase in obesity and other diet-associated health problems [3,4,5].

The COVID-19 pandemic abruptly altered Americans’ day-to-day lifestyles, including eating behavior [6,7]. One eating behavior notably altered was the consumption of FAFH [8]. In March 2020, at the onset of the COVID-19 pandemic, the amount of money spent on FAFH in the United States per month dropped by about 29%, from $37.3 billion in January 2020 to $27.1 billion in April 2020 [9]. This drop was likely due to the closure of most restaurants and the economic instability experienced by many Americans [10]. As vaccines were developed and made available and restaurants began to open, the amount spent at restaurants rebounded to $36.6 billion in March 2021 [9].

Historically, the amount a household spends on FAFH is positively correlated with disposable income [11,12]. For example, during the 2007–2009 Great Recession, disposable income decreased, and, as a result, restaurant spending decreased [2]. Another economic event, increased inflation, also typically leads to less disposable income as well as higher food prices. The interaction of higher prices and lower disposable income can also reduce the frequency and amount of money a household spends on FAFH [10].

In addition to price and disposable income, there are a few other factors that may have influenced the demand for FAFH in the post-COVID-19 period (2021 and 2022). As COVID-19 restrictions relaxed, some Americans experienced revenge spending [13,14]. This happens when an individual has been held back from spending for a variety of reasons over an extended period. If an individual has not been able to participate in an activity that they enjoy, for example, dining out, when they are able to start dining out again, they may purchase much more than they typically would. As expected, due to the lift of stay-at-home and restaurant closure policies, national trends in consumer spending on FAFH have increased post-quarantine, although they are still below those seen in 2019 (USDA 2022). In addition, in the winter of 2020/2021, the US federal government passed the Consolidated Appropriations Act of 2021 and the American Rescue Plan of 2021, which provided several economic relief measures, including Economic Impact Payments [15,16], expansion of the Child Tax Credit [17], and Emergency Rental Assistance [18]. These efforts increased households’ disposable income, which may have led to a greater demand for dining out. Finally, the US inflation rate began to increase steadily from 1.5% in February 2019 to 9.1% in June 2022 [18]. During this time period, the FAFH interest rate also increased to a lesser degree, from 2.9% in January 2019 to 7.7% in June 2022 [19], while the Food at Home interest rate increased dramatically, from 0.6% in January 2019 to 12.2% in June 2022 [19]. Based on historical observations, an increased inflation rate may reverse the observed post-pandemic spending. Finally, it was well documented that mental burden increased during the COVID-19 pandemic period beginning in the US in early 2020 [20]. High levels of mental burden, including high levels of stress, fatigue, and feelings of being overwhelmed, may have led individuals to rely on the convenience of dining out. 

Since increased consumption of dining out tends to negatively impact health status, it is important to better understand the role of the COVID-19 pandemic and the rising interest rates on dining out frequency and spending. This study examines the interaction effect between COVID-19 (before vs. after) and the inflation rate on dining out behavior as they may drive dining out behavior in different directions. We have three hypotheses:

**Hypothesis 1 (H_1_).**  *The post-COVID-19 period is positively associated with dining out frequency and spending*.

**Hypothesis 2 (H_2_).**  *The FAFH inflation rate is negatively associated with dining out frequency and spending in the post-COVID-19 period*.

**Hypothesis 3 (H_3_).**  *There is a significant interaction effect between the COVID-19 period (before vs. after) and the FAFH inflation rate on dining out frequency and spending*.

## 2. Materials and Methods

Study Design: Quasi-Experimental

### 2.1. Data Source

This study uses two data sources: survey responses from a community nutrition promotion event (hereafter Dinner Tonight) and the FAFH inflation rate data from the Bureau of Labor Statistics. Dinner Tonight is a community event intended to improve the knowledge and self-efficacy of participants in the areas of meal planning and meal preparation [21]. Providing education to promote understanding and confidence in meal planning includes highlighting the nutrition, health, and financial benefits of planning meals in advance. Dinner Tonight attendees completed a survey that asked a series of questions related to dietary behavior, household frequency of dining out, and the amount of money their household spends on dining out. Elements of the data collected via the Dinner Tonight survey are used in this study, particularly the information regarding sociodemographic characteristics, the dining out frequency per week, and the dining out spending per week. Dinner Tonight events were delivered frequently prior to the COVID-19 pandemic. However, in March 2020, group gatherings, including Dinner Tonight events, were halted due to the pandemic. Dinner Tonight events resumed in January 2021. In this study, we used the data collected by Dinner Tonight from January 2019 to June 2022. The other data source used in this study is the monthly FAFH inflation rates from January 2019 to June 2022 available from the US Bureau of Labor Statistics [19].

### 2.2. Setting

Texas A&M AgriLife Extension Service County Extension Agents are the primary implementors of the Dinner Tonight Healthy Cooking School curriculum. The Extension Service is a government-funded community education system that operates in most counties of the United States. Extension is collaboratively funded by the US Department of Agriculture, the state of operation, and the county of operation. The goal of Extension is to disseminate science-based research findings, education, and “practical information to agricultural producers, small business owners, consumers, families, and young people” [22]. County Extension Agents deliver educational programming in the communities where they work and live. As a result, Agents have a unique grasp on the community’s needs and preferences. The number of individuals who participate in Dinner Tonight events ranges from 20 to 200 people. The participation number is often a reflection of the county’s population size. During the period of this study, there were 137 Dinner Tonight events held in 50 counties in Texas. 

### 2.3. Study Population

This study examines the dining out purchasing behavior among a convenience sample collected in Texas, USA. The study population consists of members of the general population who attended their local Dinner Tonight event, either in person or virtually, and completed the Dinner Tonight Survey. The Dinner Tonight program provides County Agents with tools and resources to advertise Healthy Cooking School programs, including event invitations, save the dates, flyers, presentations, and social media graphics. It is up to County Agents to determine what will be the most effective recruitment strategy using the provided resources and any additional methods they think will be effective (e.g., newspaper articles, radio interviews). Any member of the public who registers for or attends the program is welcome to participate—there are no restrictions on who can or cannot attend a community Dinner Tonight Healthy Cooking School program.

### 2.4. Dependent Variables

Two dependent variables are used to measure dining out behavior. The first is household dining out frequency per week measured as a part of the Dinner Tonight participant survey. The second is the amount of money a household reported spending per week on dining out, measured as a part of the Dinner Tonight participant survey. Dining out only includes food purchased away from home, which means dining in a restaurant and does not include food prepared away from home and eaten in the home. Typically, the term FAFH includes food that is ”acquired” away from home, which means the food could be eaten outside of the home or acquired outside of the home but eaten in the home (e.g., delivery pizza) [2]. Since the Dinner Tonight survey asked about eating outside the home only, we use the term “dining out” throughout this article rather than FAFH. 

### 2.5. Independent Variables

We have two key independent variables: (1) the time of data collection, pre- and post-COVID-19 (COVID-19), and (2) the Food Away from Home (FAFH) inflation rate at the time of data collection (FAFH Inflation) [19]. The COVID-19 variable is dichotomous, in which the Dinner Tonight surveys administered between 1 January 2019 and 30 March 2020 represent “Before COVID-19” and the surveys administered between 1 January 2021 and 30 June 2022 represent “After COVID-19.” The Dinner Tonight program stopped collecting data immediately after the COVID-19 outbreak and resumed in January 2021. The FAFH inflation rate is the reported inflation rate that best aligns with the purchasing behavior “dining out” that is investigated in this study. Therefore, the FAFH inflation rate is used in this study as opposed to the general inflation rate. The FAFH inflation variable is the US monthly FAFH inflation rate as reported by the US Bureau of Labor Statistics at the time the Dinner Tonight survey was administered. 

### 2.6. Covariates

Five covariates are included in the multivariate models: age, gender, health status, household size, and race/ethnicity. Age is a continuous variable beginning at the age of 18 years old. Gender is reported as either female or male. Health status is reported on the Dinner Tonight Survey as “Very Good”, “Good”, Fair”, and “Poor.” In this analysis, Health Status is coded into two categories: “Good Health Status”, which includes reports of “Very Good” and “Good” health, and “Poor Health Status”, which includes reports of “Fair” and “Poor” health. Household size is the reported number of people living in the household; it is a continuous variable. Race/Ethnicity was self-reported as Hispanic, (Non-Hispanic) African American, (Non-Hispanic) White, and (Non-Hispanic) Other Race/Ethnicity. 

### 2.7. Interaction Term

An interaction term between the two independent variables (COVID-19 and FAFH Inflation) is included in two multivariate models. 

### 2.8. Statistical Analysis

The goal of this study is to evaluate the association between the COVID-19 period, FAFH inflation rate, and dining out behavior, which is measured by dining out frequency and dining out spending. We further evaluate the interaction effect between COVID-19 and the FAFH inflation rate on dining out. This study adopted descriptive and inferential statistical analyses to evaluate these relationships. In the descriptive part of this study, we summarized dining out frequency, dining out spending, FAFH inflation rate, age, and household size before and after COVID-19. Additionally, this study computed the counts and percentage of participants by sociodemographic factors, gender, health status, and race/ethnicity before and after COVID-19. Regarding the inferential statistics method, we use Zero Inflated Poisson (ZIP) regression for the model with the dependent variable of dining out frequency. Dining out frequency is count data, and it has 207 (7.2%) zero values, and the skewness test rejected the hypothesis that dining out frequency is normally distributed. Statisticians suggest that data with excess zeros should use ZIP regression [23,24,25,26,27]. We also adopted Vuong’s test to evaluate whether we should use Poisson regression or ZIP. ZIP has two parts: (1) the Poisson count model and (2) the logit model for predicting excess zeros. With respect to the total spent on dining out, the skewness test rejected the hypothesis that dining out spending is normally distributed. It also has 217 (7.8%) zero values, so this study adopted Heckman regression for inferential analysis, also called the censored regression model, to analyze skewed continuous outcomes with excess zero values [28,29]. AIC and BIC values were calculated to compare the models’ fitness. For data management, we used Python to merge the FAFH inflation rate with the data from Dinner Tonight. For statistical analysis, we used STATA for data visualization and analysis.

## 3. Results

**Figure 1:**
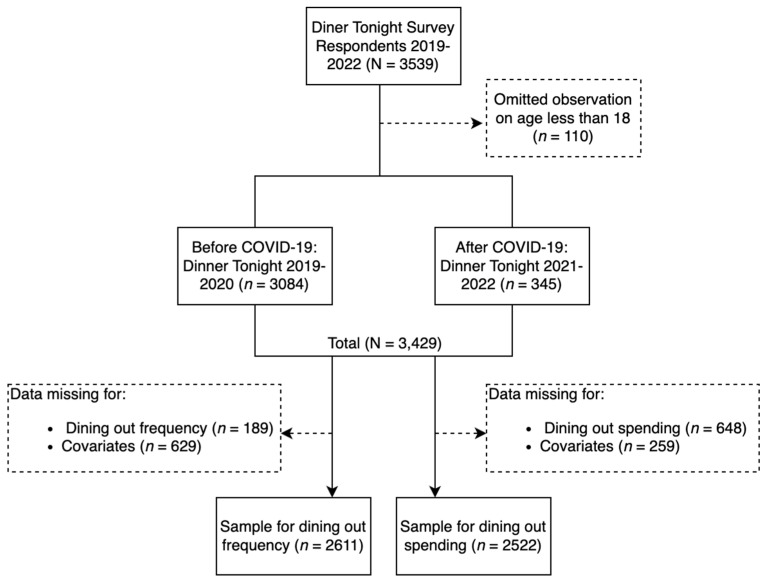
Study Population Identification Flowchart.

There were 3429 individuals who completed the Dinner Tonight survey during the study period. Of these, 2611 and 2522 have a complete data record for weekly dining out frequency and weekly dining out spending, respectively, and the covariate variables, Figure 1. The household average dining out frequency per week was 3.4 before COVID-19 and 3.5 after COVID-19, Table 1. The average dining out spending per week was $63.90 before COVID-19 and $82.20 after COVID-19. The survey respondents were primarily females, of Non-Hispanic White or Hispanic race/ethnicity, and reported being in good health. The average age of the respondents was in the early to middle 50s. 

To examine the relationship between the pandemic and the role of FAFH inflation on dining out frequency, refer to models 1 and 3, Table 2. Model 1 has two components (1.a and 1.b). Model 1.a only includes individuals who reported dining out at least once per week. Model 1.b compares households that reported dining out at least once per week to those that reported no dining out per week. Looking at Model 1.a, dining out frequency increased after the COVID-19 pandemic, while dining out frequency decreased as the FAFH inflation rate increased. Several covariate factors such as increased age, being female vs. male, having good health status vs. poor health status, and increased household size were associated with decreased dining out frequency. Model 1.b shows the relationship between independent variables and households that reported no dining out in the past week. Neither the pandemic nor the FAFH inflation rate significantly contributed to the outcome of no dining out in the past week. Looking at the covariate factors, those with a larger household size were less likely to acquire no dining out per week, while those who were African American or Other Race/Ethnicity were more likely to report no dining out per week.

**Figure 2:**
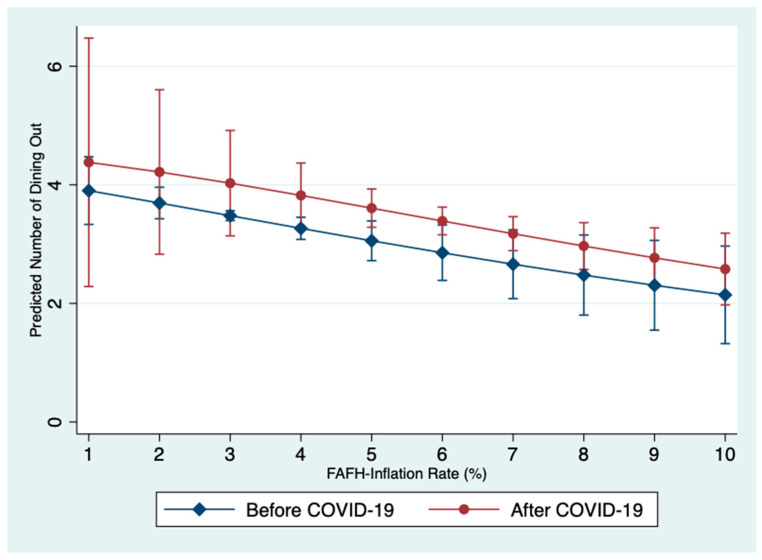
Predicted Weekly Dining Out Frequency Before and After COVID-19 as Food Away from Home (FAFH) Inflation Rate Fluctuates. Note: Figure 2 is based on Model 1 in Table 2.

To further examine the role of the pandemic and FAFH inflation rate on dining out frequency, an interaction variable (COVID-19*FAFH inflation rate) was added to Model 1; see Model 3. Model 3 has two components (Model 3.a and Model 3.b) and included all the variables included in Model 1. The difference in these models is the addition of a variable that represents the interaction between COVID-19 and the FAFH inflation rate. In the area of dining out frequency, there is no significant interaction effect between COVID-19 and the FAFH inflation rate. When comparing the fit of Model 1 to Model 3, we see that Model 1 has relatively smaller AIC and BIC values compared to Model 3. This indicates that the results presented in Model 1 are more robust than the results in Model 3. The relationship between the predicted dining out frequency and FAFH interest rate percentage are shown in Figure 2; these figures are based on model 1.a and therefore adjusted for all of the covariates included in model 1.a. Since interaction was not significant, we do expect parallel lines for the predicted numbers of dining out per week for different inflation rates pre- and post-COVID-19. However, we want to mention that the precision of the predictions vary (as indicated by the widths of 95% prediction intervals) depending on the observed counts in each scenario in the dataset.

Model 2 examines the factors associated with the amount of money a household spent on dining out in the past week. There was not a significant change in the amount of money spent on dining out pre- and post-pandemic. There also was not a significant relationship between the amount spent on dining out and the FAFH inflation rate. Weekly spending increased as household size increased, while weekly spending decreased as age increased. Weekly spending was lower for those who were female compared to male, in good health compared to poor health, and Hispanic, African American, or Other Race Ethnicity compared to White respondents. 

To further examine the role of the pandemic and the FAFH inflation rate on the amount of money a household spent on dining out in the past week, an interaction variable (COVID-19*FAFH inflation rate) was added to Model 2; see Model 4. There is no significant interaction between the COVID-19 (before and after) period and the fluctuating inflation rate. When comparing the fit of Model 2 to Model 4, we see that Model 2 has a relatively smaller AIC and BIC value compared to Model 4. This indicates that the results presented in Model 2 are more robust than the results in Model 4. 

Study findings were partially consistent with Hypothesis 1, “The post COVID-19 period is positively associated with dining out frequency and spending.” After the COVID-19 pandemic, the expected log count of dining out per week increased by 0.19, Table 2, Model 1.a. However, the post COVID-19 period did not significantly contribute to a change in the amount of money spent on dining out, Table 2, Model 2. Study findings were inconsistent with Hypothesis 2: “The inflation rate is negatively associated with dining out frequency and spending in the post COVID-19 period” regardless of timing (pre- or post-COVID-19 pandemic). A one-unit increase in the inflation rate was associated with a decrease in expected log count of dining out per week, a reduction of 0.07, Table 2, Model 1.a. However, the fluctuating inflation rate pre- and post-COVID-19 was not associated with the amount spent on dining out, Table 2, Model 2. Study findings were inconsistent with Hypothesis 3: “There is a significant interaction effect between COVID-19 and the inflation rate on dining out frequency and spending.” No significant interaction effect was found, Table 1, Models 3 and 4. 

## 4. Discussion

This study examined the relationships between the period of the COVID-19 pandemic, the US FAFH inflation rate, and dining out household weekly frequency and spending as well as the interactive effect between COVID-19 and the FAFH inflation rate on dining out behaviors. First, participants’ dining out frequency increased after the COVID-19 pandemic, which is consistent with previous studies that the post-COVID-19 period was associated with revenge consumption [13,14]. An increase in dining out frequency may also be explained by an increase in disposable income obtained via federal stimulus programs [15,16,17,18]. The increase in dining out frequency may also be due, in part, to the accelerated increase in the Food at Home interest rate [19]. Purchasing prepared food away from home is frequently more expensive than food made at home [2]. However, as the Food at Home interest rate increased, the cost of food at home relative to food purchased when dining out may have narrowed, encouraging individuals to dine out more. Further research to identify motivators of dining out frequency post-pandemic is warranted. 

Second, the FAFH inflation rate was negatively associated with participants’ dining out frequency, which is consistent with previous research findings that the inflation rate is negatively associated with consumption behaviors [12,30]. The time, before or after the COVID-19 pandemic, did not modify this relationship. Further research to examine whether the findings presented here are consistent in a larger, more geographically diverse sample are warranted. 

If the experience of the general American population is similar to those included in this study, the findings raise a public health concern. The health community has been concerned about the steady rise in dining out frequency in the past 35 years because of the negative relationship between dining out frequency and diet quality [3,4,5]. Our results indicate that the pandemic exacerbated this increase in dining out frequency. 

This study has unique strengths and limitations. One strength is that, to our knowledge, this is one of the first studies to explore the influence of the pandemic on dining out behaviors [9,31]. In addition, the data used in this study included both pre- and post-pandemic data. Finally, the study sample was relatively racially and ethnically diverse. There are a few limitations. First, the study population was limited to those in Texas and therefore may not be generalizable to the US population. Second, the study period is also limited to a unique time in US history and therefore not representative of future dining out behavior. Third, the data available did not include factors that may have been important to capture such as feelings of stress, fatigue, and revenge spending or measures of receipt of federal recovery benefits [15,16,17,18,20]. Finally, the study population consisted of people interested in learning about meal preparation strategies. These individuals may have different dining out behaviors than the general population. As our survey’s main purpose was to evaluate Dinner Tonight’s effect on participants’ dietary behavior, it does not include information on income/wage and prices, which may require future research to include this information.

## 5. Conclusions

This analysis found that in the post-COVID-19 period (2021 and 2022), demand for dining out frequency increased. Additional research to better understand the reasoning for this finding is warranted. Researchers are encouraged to investigate the factors that have led the American public to increasingly rely on dining out and the impact of that reliance on health outcomes.

## Figures and Tables

**Table 1:** Household Dining Out Frequency and Spending, FAFH Inflation Rate, and Sociodemographic Descriptive Statistics Before and After the COVID-19 Pandemic.

	Before COVID-19 *n* = 3084 (90%)		After COVID-19 *n* = 345 (10%)	
Variable	*n* (%)	Mean (Std. Dev)	*n* (%)	Mean (Std. Dev)
Weekly Dining Out Frequency (#)		3.4 (3.2)		3.5 (3.1)
Weekly Dining Out Spending ($)		63.9 (62.3)		82.2 (77.7)
Monthly FAFH Inflation Rate (%)		3.07 (0.1)		6.0 (1.0)
Age		53.0 (16.5)		52.9 (15.6)
Gender				
Female	2295(78.8)		241(71.7)	
Male	616 (21.2)		95 (28.3)	
Health Status				
Poor	554 (18.8)		71 (20.9)	
Good	2391 (81.2%)		269 (79.1)	
Household Size		2.7 (1.6)		2.6 (1.3)
Race/Ethnicity:				
African American	242 (8.2)		41 (12.3)	
Hispanic	1107 (37.5)		74 (22.2)	
Other Race/Ethnicity	132 (4.5)		10 (3.0)	
White	1468 (49.8)		209 (62.6)	

**Table 2:** The Influence of the COVID-19 Pandemic and FAFH Inflation on Household Dining Out Frequency and Spending.

	Model 1 (*n* = 2611)		Model 2 (*n* = 2522)	Model 3 (*n* = 2611)		Model 4 (*n* = 2522)
Variables	Model 1. a Dining out frequency for those who dined out at least one time per week	Model 1.b No dining out in the past week	Money Spent on dining out in the past week	Model 3.a Dining out frequency for those who dined out at least one time per week with interaction variable	Model 3.b No dining out in the past week with interaction variable	Money spent on dining out in the past week including an interaction variable
After the COVID-19 Pandemic	0.19 *	0.82	−0.21	0.19 *	−2.82	1.03
	(0.09)	(1.03)	(0.23)	(0.09)	(3.61)	(0.75)
FAFH Inflation Rate	−0.07 **	−0.34	0.08	−0.07 **	−1.23	0.38 *
	(0.03)	(0.38)	(0.07)	(0.03)	(0.92)	(0.19)
Age	−0.01 ***	0.01	−0.57 ***	−0.01 ***	0.01	−0.57 ***
	(0.00)	(0.01)	(0.08)	(0.00)	(0.01)	(0.08)
Gender: Female (ref: Male)	−0.22 ***	−0.56	−11.18 ***	−0.22 ***	−0.55	−11.17 ***
	(0.02)	(0.29)	(2.88)	(0.02)	(0.29)	(2.88)
Good Health Status (ref: Poor Health Status)	−0.07 *	−0.03	−7.85 *	−0.07 *	−0.02	−7.85 *
	(0.03)	(0.36)	(3.26)	(0.03)	(0.36)	(3.26)
Household Size	−0.03 ***	−0.81 ***	6.86 ***	−0.03 ***	−0.77 ***	6.86 ***
	(0.01)	(0.24)	(0.89)	(0.01)	(0.23)	(0.89)
Race/Ethnicity (ref: White)						
Hispanic	0.02	−0.50	−9.43 **	0.02	−0.53	−9.42 **
	(0.03)	(0.43)	(2.90)	(0.03)	(0.42)	(2.90)
African American	−0.07	0.79 *	−15.42 ***	−0.07	0.75	−15.41 ***
	(0.04)	(0.39)	(4.62)	(0.04)	(0.39)	(4.62)
Other Race/Ethnicity	−0.11	1.25 **	−20.02 **	−0.11	1.27 **	−20.02 **
	(0.06)	(0.46)	(6.19)	(0.06)	(0.45)	(6.19)
COVID−19* FAFH Inflation Rate					1.05	−0.35
					(0.99)	(0.20)
Constant	2.23 ***	−0.64	98.92 ***	2.23 ***	2.07	−0.33
	(0.11)	(1.48)	(7.56)	(0.11)	(2.94)	(0.57)
athrho			−0.04			−0.04
			(0.13)			(0.13)
lnsigma			4.12 ***			4.12 ***
			(0.01)			(0.01)
Vuong		4.38 ***				
Model Fit Statistics:						
AIC		13,249.22	31,156.94		13,250.13	31,155.9
BIC		13,366.57	31,235.74		13,373.34	31,240.76

Notes: ref is reference; standard errors in parentheses. Vuong test is significantly positive (z = 4.38 ***), which shows Zero Inflated Poisson model (model 1) is favored over the simple Poisson model. FAFH Inflation Rate means Consumer Price Index percentage change in Food Away From Home (https://www.bls.gov/opub/ted/2022/prices-for-food-at-home-up-13-5-percent-for-year-ended-august-2022.htm) (accessed on 5 December 2022). *** *p* < 0.001, ** *p* < 0.01, * *p* < 0.05.

## Data Availability

Not applicable.

## References

[1] Lachat C., Nago E., Verstraeten R., Roberfroid D., Van Camp J., Kolsteren P. (2012). Eating out of home and its association with dietary intake: A systematic review of the evidence. Obes. Rev..

[2] Saksena M.J., Okrent A.M., Anekwe T.D., Cho C., Dicken C., Effland A., Elitzak H., Guthrie J., Hamrick K.S., Hyman J. (2018). America’s Eating Habits: Food Away from Home.

[3] Cohen D.A., Story M. (2014). Mitigating the health risks of dining out: The need for standardized portion sizes in restaurants. Am. J. Public Health.

[4] Oh C., Kim H.-S., No J.-K. (2015). Impact of dining out on nutritional intake and metabolic syndrome risk factors: Data from the 2011 Korean National Health and Nutrition Examination Survey. Br. J. Nutr..

[5] Wright B., Bragge P. (2018). Interventions to promote healthy eating choices when dining out: A systematic review of reviews. Br. J. Health Psychol..

[6] Ammar A., Brach M., Trabelsi K., Chtourou H., Boukhris O., Masmoudi L., Bouaziz B., Bentlage E., How D., Ahmed M. (2020). Effects of COVID-19 home confinement on eating behavior and physical activity: Results of the ECLB-COVID19 international online survey. Nutrients.

[7] Scarmozzino F., Visioli F. (2020). COVID-19 and the subsequent lockdown modified dietary habits of almost half the population in an Italian sample. Foods.

[8] Yang Y., Liu H., Chen X. (2020). COVID-19 and restaurant demand: Early effects of the pandemic and stay-at-home orders. Int. J. Contemp. Hosp. Manag..

[9] Marchesi K., McLaughlin P.W. (2022). COVID-19 Working Paper: The Impact of COVID-19 Pandemic on Food-Away-From-Home Spending.

[10] Kumcu A., Kaufman P.R. (2011). Food Spending Adjustments during Recessionary Times. https://www.ers.usda.gov/amber-waves/2011/september/food-spending/.

[11] Schnepf R. Consumers and Food Price Inflation. DIANE Publishing: 2011. https://sgp.fas.org/crs/misc/R40545.pdf.

[12] Schnepf R.D., Richardson J. Consumers and Food Price Inflation. https://www.everycrsreport.com/files/20091104_R40545_01aee0c4d86f1b107582200c5d61d6fd2ead5d43.pdf.

[13] Gupta A.S., Mukherjee J. (2022). Long-term changes in consumers’ shopping behavior post-pandemic: An exploratory study. Int. J. Retail Distrib. Manag..

[14] Park I., Lee J., Lee D., Lee C., Chung W.Y. (2022). Changes in consumption patterns during the COVID-19 pandemic: Analyzing the revenge spending motivations of different emotional groups. J. Retail. Consum. Serv..

[15] Murphy D. (2021). Economic Impact Payments.

[16] Economic Impact Payments. https://home.treasury.gov/policy-issues/coronavirus/assistance-for-american-families-and-workers/economic-impact-payments.

[17] Hamilton L., Roll S., Despard M., Maag E., Chun Y., Brugger L., Grinstein-Weiss M. (2022). The impacts of the 2021 expanded child tax credit on family employment, nutrition, and financial well-being: Findings from the Social Policy Institute’s Child Tax Credit Panel (Wave 2). Glob. Econ. Dev. Brook..

[18] USDT (2020). Emergency Rental Assistance Program. https://home.treasury.gov/policy-issues/coronavirus/assistance-for-state-local-and-tribal-governments/emergency-rental-assistance-program.

[19] U.S Bureau of Labor Statistics. 12-Month Percentage Change in Consumer Prices for Selected Food Items, January 1968–August 2022. https://www.bls.gov/opub/ted/2022/prices-for-food-at-home-up-13-5-percent-for-year-ended-august-2022.htm.

[20] Kunzler A.M., Röthke N., Günthner L., Stoffers-Winterling J., Tüscher O., Coenen M., Rehfuess E., Schwarzer G., Binder H., Schmucker C. (2021). Mental burden and its risk and protective factors during the early phase of the SARS-CoV-2 pandemic: Systematic review and meta-analyses. Glob. Health.

[21] Texas A&M Agrilife Extension Dinner Tonight. https://dinnertonight.tamu.edu/.

[22] USDA National Institute of Food and Agriculture. https://www.nifa.usda.gov/about-nifa/how-we-work/extension.

[23] Afifi A.A., Kotlerman J.B., Ettner S.L., Cowan M. (2007). Methods for improving regression analysis for skewed continuous or counted responses. Annu. Rev. Public Health.

[24] Lambert D. (1992). Zero-inflated Poisson regression, with an application to defects in manufacturing. Technometrics.

[25] Loeys T., Moerkerke B., De Smet O., Buysse A. (2012). The analysis of zero-inflated count data: Beyond zero-inflated Poisson regression. Br. J. Math. Stat. Psychol..

[26] Mouatassim Y., Ezzahid E.H. (2012). Poisson regression and Zero-inflated Poisson regression: Application to private health insurance data. Eur. Actuar. J..

[27] Yusuf O., Bello T., Gureje O. (2017). Zero inflated poisson and zero inflated negative binomial models with application to number of falls in the elderly. Biostat. Biom. Open Access J..

[28] Boulton A.J., Williford A. (2018). Analyzing skewed continuous outcomes with many zeros: A tutorial for social work and youth prevention science researchers. J. Soc. Soc. Work Res..

[29] Tobin J. (1958). Estimation of relationships for limited dependent variables. Econom. J. Econom. Soc..

[30] Lee A., Mhurchu C.N., Sacks G., Swinburn B., Snowdon W., Vandevijvere S., Hawkes C., L’Abbé M., Rayner M., Sanders D. (2013). Monitoring the price and affordability of foods and diets globally. Obes. Rev..

[31] Janssen M., Chang B.P., Hristov H., Pravst I., Profeta A., Millard J. (2021). Changes in food consumption during the COVID-19 pandemic: Analysis of consumer survey data from the first lockdown period in Denmark, Germany, and Slovenia. Front. Nutr..

